# Quality assessment of different brands of atorvastatin tablets available in Riyadh, Saudi Arabia

**DOI:** 10.1186/s40360-022-00598-y

**Published:** 2022-09-13

**Authors:** Ali AlMuhsin, Abdul Ahad, Yousef A. Bin Jardan, Mohammad Raish, Ajaz Ahmad, Khalid M. Alkharfy, Fahad I. Al-Jenoobi

**Affiliations:** 1 Department of Pharmaceutics, College of Pharmacy, King Saud University, P.O. Box 2457, Riyadh, 11451 Saudi Arabia; 2 Department of Inspection support, Saudi Food and Drug Authority, Riyadh, Saudi Arabia; 3 Department of Clinical Pharmacy, College of Pharmacy, King Saud University, Riyadh, 11451 Saudi Arabia

**Keywords:** Atorvastatin, Dissolution, Friability test, HPLC, Tablets, Weight variations

## Abstract

**Background:**

Hypolipidemic agents have been shown to be helpful in the primary and secondary prevention of cardiovascular disease. Most often, statins are prescribed to treat hyperlipidemia. There are a number of statins available in the market today, but atorvastatin is the most widely prescribed. It is essential that the drugs should have the appropriate amount of active pharmaceutical ingredient and meet the necessary physical properties. The main purpose of the study was to evaluate the quality of different marketed brands of atorvastatin calcium tablets available in Saudi Arabia.

**Methods:**

In this study, innovator product coded as (AS-1) and five generics brands (coded as AS-2 to AS-6) of atorvastatin tablets 20 mg available in Saudi Arabia were evaluated for *in vitro* dissolution test, weight variations, friability and hardness tests. The analysis of drug was carried out by “high-performance liquid chromatography” (HPLC) method using C_18_ column (4.6 × 150 mm, 5 μm). The mobile phase was consisted of acetonitrile and HPLC water (pH 2.1, adjusted with orthophosphoric acid) in ratio of 52:48 v/v, the flow rate was 1.0 ml/min. Atorvastatin was detected at a wavelength of 254 nm.

**Results:**

According to the results of the dissolution study, the investigated products released more than 90% of atorvastatin in 15 min. Within 60 min, the brands AS-1, AS-3, AS-5, and AS-6 depicted nearly 100% atorvastatin release, while the brand AS-2 displayed 91.69% drug release. According to our findings, the investigated atorvastatin innovator (AS-1) and generic brands such as AS-2 to AS-6 were of good pharmaceutical quality.

**Conclusions:**

All generic brands of atorvastatin tablets available in the Saudi Arabian market met the pharmacopoeia's consistency checks such as weight variation, friability, hardness and in vitro dissolution. Hence, focusing on their in vitro release properties, it was determined that these brands could be used interchangeably.

## Background

The world markets for lipid-lowering drugs are largely dominated by statins. Of these, atorvastatin is the most commonly prescribed [1]. The drug is on the top of list of highest-selling drug all the time [2]. Others drug belongs to class called statin includes fluvastatin, lovastatin, pravastatin, rosuvastatin, simvastatin and pitavastatin [3, 4].

**Fig. 1:**
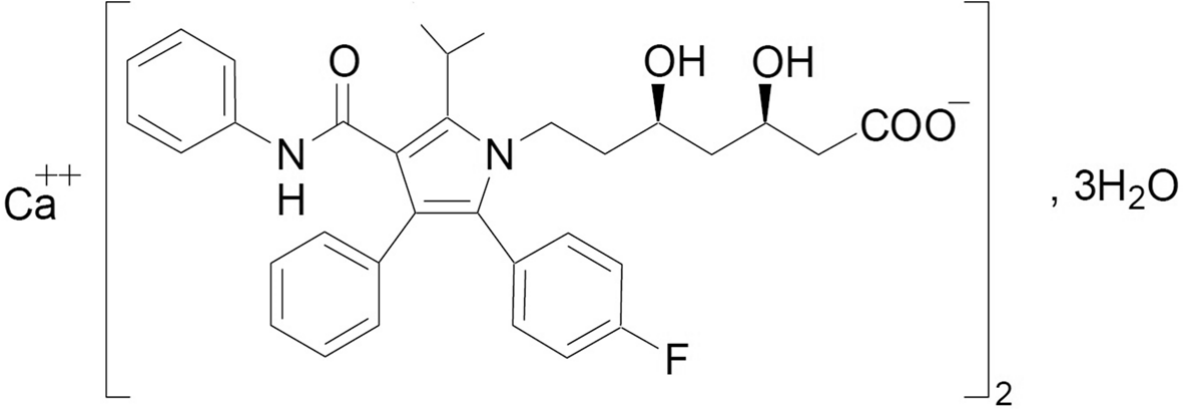
Chemical structure of atorvastatin calcium

Atorvastatin calcium (Fig. 1) has molecular weight of 1155.363 g/mol and the chemical formula is C_66_H_68_CaF_2_N_4_O_10_ [5–7]. It is freely soluble in methanol, slightly soluble in ethanol, and very slightly soluble in distilled water, phosphate buffer with pH 7.4, and acetonitrile [6, 8, 9]. Due to its low soluble and high permeable it can be classified under class II [10].

The brand drug was approved by “the United States Food and Drug Administration (USFDA)” in 2001 under name Lipitor^TM^ (atorvastatin calcium) as film coated tablets for company Pfizer Ireland Pharmaceuticals and available in the market in four strengths (10 mg, 20 mg, 40 mg and 80 mg) [11]. The atorvastatin are indicated in patients with hypercholesterolemia, heterozygous familial hypercholesterolemia and prevention of cardiovascular events in patients at high risk of a first cardiovascular event [12, 13].

These lipid-lowering medicines reducing plasma cholesterol by inhibition of 3-hydroxy-3-methylglutaryl-coenzyme A (HMG-CoA) reductase, this enzyme is acting as catalyst in mevalonate pathways that lead to form several compound in the body like cholesterols [5, 14]. There are other activity for statins which are antitumor sensitizing agent by control of tumor initiation, growth, and metastasis [12, 15].

The oral dose of 10 mg atorvastatin contributes a bioavailability of around 14%, and it is predominantly metabolized in the liver by the “cytochrome P450 (CYP)” isoenzyme CYP3A4 in two active forms, 2-hydroxy-atorvastatin acid and 4-hydroxy-atorvastatin acid [5, 16, 17]. It has a poor absolute bioavailability because of presystemic clearance in the gastrointestinal mucosa and/or first-pass metabolism in the liver, which is its main site of action. It binds to plasma proteins to the extent of 98 percent [18]. The half-life of atorvastatin in the blood is around 14 h, however attributed to the impact of active metabolites, the inhibitory effect for HMG-CoA reductase is between 20 to 30 h. Atorvastatin is excreted mainly as metabolites in the bile [18, 19].

Many studies have been published on the in vitro drug release of various categories of commercial tablets in different countries [20–25]. But there is a dearth of literature on such information of atorvastatin marketed in Saudi Arabia. Hence, locally available atorvastatin tablets were chosen for the assessment with particular focus on the dissolution property, because of its significance in forecasting medication bioavailability and product quality.

Previously, Ulla et al. (2018) assessed the consistency of atorvastatin calcium tablet generic brands obtainable in the Asir region of Saudi Arabia. In addition to the innovator product, three generic brands were mentioned in the analysis and were subjected to disintegration test, in-vitro dissolution (medium pH 1.2 HCl, 37 ± 0.5 °C and 100 rpm) and quality control tests such as hardness, friability, and assay using UV spectrophotometer at wavelength 246 nm. The findings of in vitro drug dissolution profiles of both innovator and generic brands indicate that, greater than 90% of the drug is released during one hour [26].

In another study, Tariq et al. (2014) correlated the in vitro bioequivalence of six generic atorvastatin brands available in Pakistan with the innovator brand. For dissolution experiment, phosphate buffer pH 6.8 at 37 0.5°C and 75 rpm was used as the medium. By measuring the similarity factors f1 and f2, the findings suggest no similarity in five of the six generics investigated. The drug content was well within the appropriate range for all of the brands. Considerable variances were noted by the generics brands through the evaluation of other quality control examination [27]. Antecedently, Popy et al. published an assessment of three atorvastatin generics present in Bangladesh in relation to the innovator product, which included an in vitro bioequivalence analysis in three different media (pH 1.2, pH 4.5, and pH 6.8). The findings demonstrate that all of the brands displayed similarity in pH 6.8 without measuring the similarity factor f2, because all of the generics brands released over than 85% of the drug within 15 minutes. By measuring the similarity factor f2, the findings of dissolution study in medium having pH 4.5 indicate similarity, but at dissolution study done in medium having pH 1.2, the similarity is lost. The investigation also included the characterization of crushing strength, friability, and weight uniformity, and found no variations between innovator and generics products [28]. Earlier, Oliveira et al. (2012) developed method to compare innovator product of atorvastatin with one generic product available in Brazil [29].

In 2012, Akinleye et al. examined into the quality of three products, one of which was an atorvastatin calcium tablet brand sold in Lagos, Nigeria. The following tests were performed: active ingredient assay, in-vitro dissolution test, uniformity of weight, hardness, friability, and disintegration test. The dissolution medium was maintained at 37 ± 0.5°C and 50 rpm. Except for the in-vitro dissolution test, all of the findings were acceptable. The test resulted in the failure of one generic product. At 60 minutes, it had only released 70% of its label assertion. The second generic delivered about 75% of the label claim in 45 minutes [30].

The effectiveness of pharmaceutical formulations systems is mainly determined by their formulation and manufacturing procedures, hence dosage system quality might fluctuate. Previous studies exhibited that some brands of atorvastatin products failed to meet pharmacopeial standards [30–33]. Therefore, it is imperative for researchers to conduct the independent bioequivalence assessment of marketed drug products in this environment. Product performance tests are designed to assess product performance and in many cases relate to dissolution [34, 35]. Consequently, present investigation was directed to evaluate the drug product performance of AS-1 (innovator) and five generic products (AS-2, AS-3, AS-4, AS-5, and AS-6) available in Saudi Arabia. Further, friability, hardness and tablet weight variations were also considered [23].

## Materials and methods

### Materials

Acetonitrile was purchased from Sigma- St. Louis, MO, USA. Sodium hydroxide was obtained from Merck-Darmstadt, Germany. Potassium dihydrogen phosphate and methanol were procured from Riedel-De Haen AG- Seeize, Germany, and Panreac- Barcelona, Spain, respectively.

### Sampled drug products

All brands of atorvastatin tablets (20 mg) were purchased for testing from retail pharmacies in Riyadh, Saudi Arabia with their original packaging and were within their expiration dates. The products were coded as AS-1 (innovator), AS-2, AS-3, AS-4, AS-5 and AS-6. All generic products (AS-2 to AS-6) were compared against the innovator product AS-1.

### Identification test

The identification of the correct active pharmaceutical ingredient in the tablets dosage form was confirmed by HPLC analytical technique. The experiment was carried out by comparing the sample and standard solution's peak retention times [22].

### Weight variation

The weight variation of the tablets was determined by Sartorius BP analytical balance, Model BP61S. The twenty tablets of each product being individually weighted and the standard deviation recorded.

### Friability test

In this study, the tablet friability tester (Model EF-2 Electrolab) was used to perform the friability test. The variation between the weights of the tablets before and after the test was measured. The friability tester timer is set to 4 minutes and the speed is set to 25 rpm.

### Hardness test

Hardness test was done using Erweka hardness tester TBH 28, an average of hardness test results was obtained and standard deviation calculated.

### Dissolution test

The dissolution medium (0.05 M phosphate buffer) was prepared by using potassium dihydrogen phosphate in purified water and pH 6.8 was adjusted with the help of sodium hydroxide and pH was monitored by digital pH meter (Toledo seven easy^TM^).

The dissolution test was conducted using “United States Pharmacopeia” (USP) 2 sotax dissolution system equipped with SAM xtend (sotax) sample collector and CP xtend (sotax) pump. The dissolution apparatus was operated by Winsotax plus (dissolution testing and management) ver. 2.57.2.4129.3958. The selection of an appropriate dissolution medium is crucial for unbiased evaluation. Atorvastatin calcium is an official component of the Indian Pharmacopoeia, which specifies phosphate buffer as a dissolution medium [36]. Hence, dissolution was performed in 900 ml phosphate buffer of pH 6.8 at 37 ± 0.5 °C at 75 rpm [7, 27, 28]. An auto sampler was coupled to dissolution apparatus was programed to withdraw and replace 1 ml of the dissolution media at 5, 10, 15, 30 and 60 min.

### Analysis by HPLC

The HPLC system consisted of “Shimadzùs CTO-20A Prominence column oven”, “SPD 20A UV/VIS detector”, “SIL-20A auto-sampler”, “CBM-20A communication Bus Module”, “LC-20AD solvent delivery pump” and “DGU-20A5 degasser” was used for the assay of atorvastatin. The data were acquired and processed with “Shimadzu LC Solution software”. The “Nucleodur C_18_ column (4.6 × 150 mm, 5 μm; Macherey-Nagel, Duren, Germany)” was used. The mobile phase consisted of acetonitrile and HPLC water (pH 2.1, adjusted with orthophosphoric acid) in ratio of 52:48 v/v, filtered through a 0.45 μm Millipore filter and degassed prior to use. The flow rate was 1.0 ml/min. Atorvastatin was detected at a wavelength of 254 nm [30]. Injection volume was 25 μl and total run time was 10 min, and column temperature was maintained at ambient.

## Results and discussion

In order to minimize the incidence of getting low-quality medicines in the supply chain, it is important to evaluate the quality output of drugs that are available in markets. The consistency of commercially available brands of atorvastatin tablets in Saudi Arabia was determined in this experiment. Furthermore, the quality control tests were performed on all atorvastatin tablets brands available in the market to ascertain their dissolution rate as well as other quality attributes such as tablets weight variation, friability, and hardness. Atorvastatin tablets of all brands were found uniform in color, size and shape. Tablets from all brands did not showed any sign of crack or break (Table 1). The packing and labeling information of the different brands of atorvastatin tablets are tabulated in Table 2.

**Table 1:** Physical characteristics of the tablets

Product code	Uniformity of colour	Uniformity of size	Uniformity of shape	Surface spot or contamination	Breaks, and cracks
AS-1	✓	✓	✓	X	X
AS-2	✓	✓	✓	X	X
AS-3	✓	✓	✓	X	X
AS-4	✓	✓	✓	X	X
AS-5	✓	✓	✓	X	X
AS-6	✓	✓	✓	X	X

**Table 2:** Packing and labeling information for the different brands

Product code	Medicine strength (mg/tablet)	Dosage statement	Batch/Lot no.	Storage condition	Manufacturing date	Expiry date
AS-1	✓	✓	✓	✓	05/2016	04/2019
AS-2	✓	✓	✓	✓	11/2016	11/2018
AS-3	✓	✓	✓	✓	01/2016	01/2019
AS-4	✓	✓	✓	✓	03/2017	12/2018
AS-5	✓	✓	✓	✓	10/2017	10/2019
AS-6	✓	✓	✓	✓	11/2017	11/2020

### Identification test

**Fig. 2:**
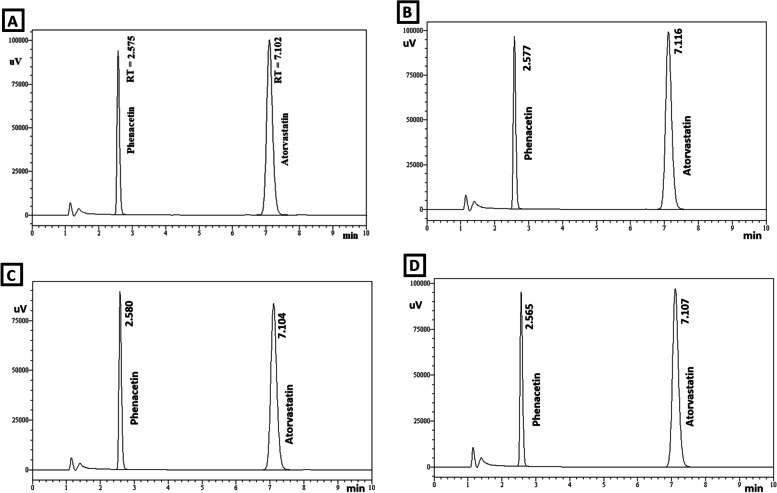
Peak retention time of (**A**) atorvastatin standard sample and samples prepared from tablets (**B**) AS-1 (**C**) AS-2 (**D**) AS-3

**Fig. 3:**
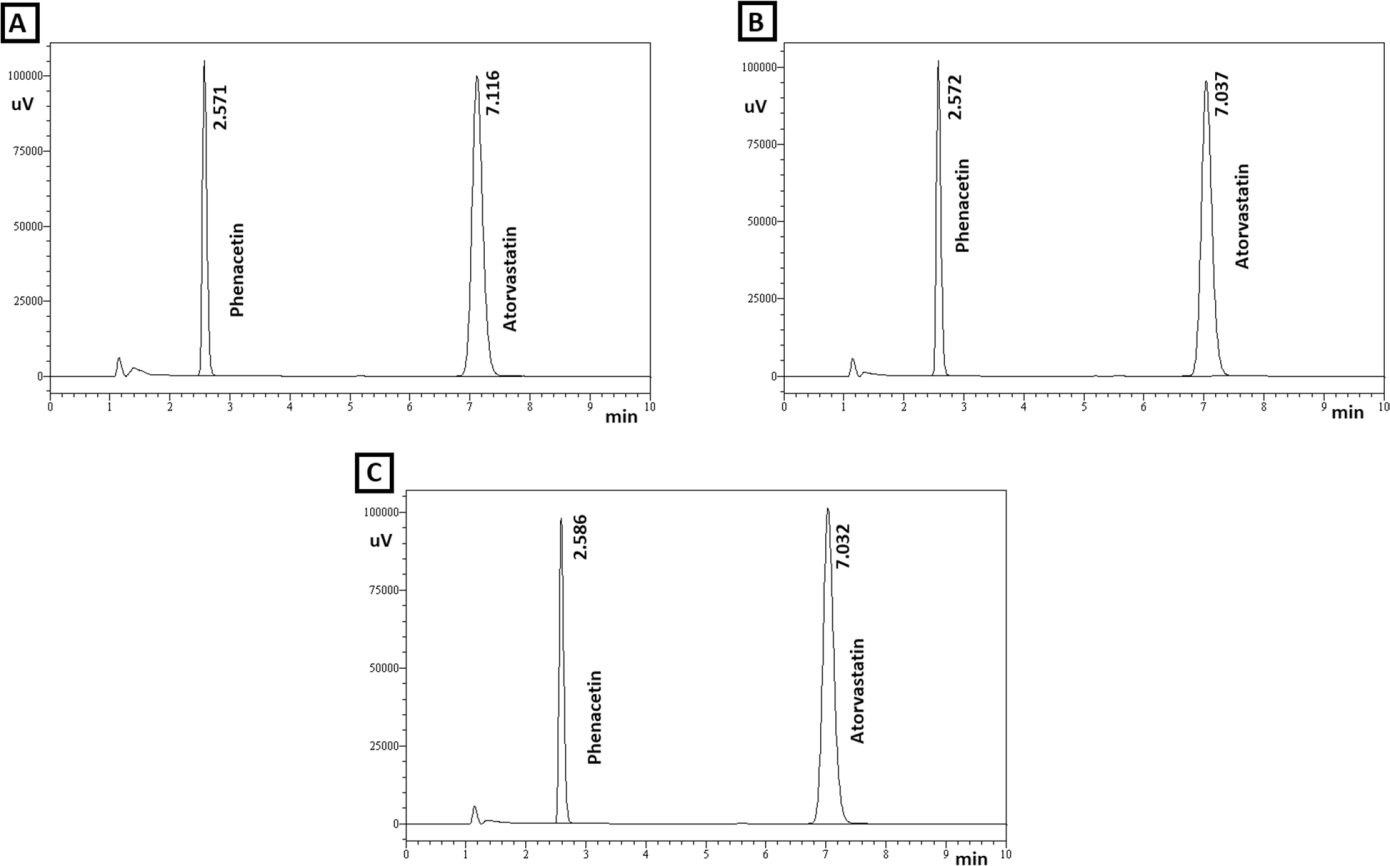
Peak retention time of atorvastatin sample prepared from tablets (**A**) AS-4 (**B**) AS-5 and (**C**) AS-6

Figures 2 and 3 indicates that the peak retention time of atorvastatin in the samples prepared from tablets ranged from 7.032 min to 7.116 min and the peak retention time of internal standard (phenacetin, final concentration 4 μg/ml) ranged from 2.565 min to 2.586 min. The peak retention time of internal standard and atorvastatin standard sample was found to be 2.575 min and 7.102 min respectively (Fig. 2 A). The internal standard is employed to improve the precision of quantitative analysis. All of the samples prepared from atorvastatin tablets products (AS-2 to AS-6) demonstrated atorvastatin peak retention times that were similar to the atorvastatin standard samples. This supported the authenticity of the atorvastatin contained in the dosage form [22].

### Weight variation

As shown in Table 3, all of the atorvastatin-containing tablets tested were well below the USP's 10% weight variation limit and succeeded the weight variation test. The weight uniformity revealed that all the investigated atorvastatin brands contained the labeled amount of drug and ensuring dosage uniformity.

**Table 3:** Evaluation of weight variation and hardness of different brands of atorvastatin tablets

Product code	AS-1	AS-2	AS-3	AS-4	AS-5	AS-6
**Average weight (mg)**	304.25	305.75	253.70	213.15	256.70	308.40
**SD**	3.06	2.57	2.79	2.56	2.70	2.30
**Average hardness (kgf)**	17.10	9.23	16.54	14.38	17.42	6.95
**SD**	0.98	0.66	0.96	0.22	1.70	0.24

### Friability test

Friability test is employed to assess the tablets resistance to abrasion. Friability is at the present incorporated in the USP, 1995 as a compendia test [25]. The compendia requirement for friability test is 1%. In our study, the outcomes of friability test were found to be <1% for all the investigated brands of atorvastatin.

### Hardness

Hardness test demonstrated that each of the brands is sturdy sufficient to endure the pressure without having to lose any of the tablet components throughout the handling and packaging process. According to our findings, the generic product (AS-3 and AS-5) demonstrated hardness values similar to the innovator product (Table 3). Although, the brand AS-2, AS-4, and AS-6 exhibited lower hardness, this could be the reason for their relatively faster disintegration than the innovator product (Table 3).

### HPLC analysis of atorvastatin

**Fig. 4:**
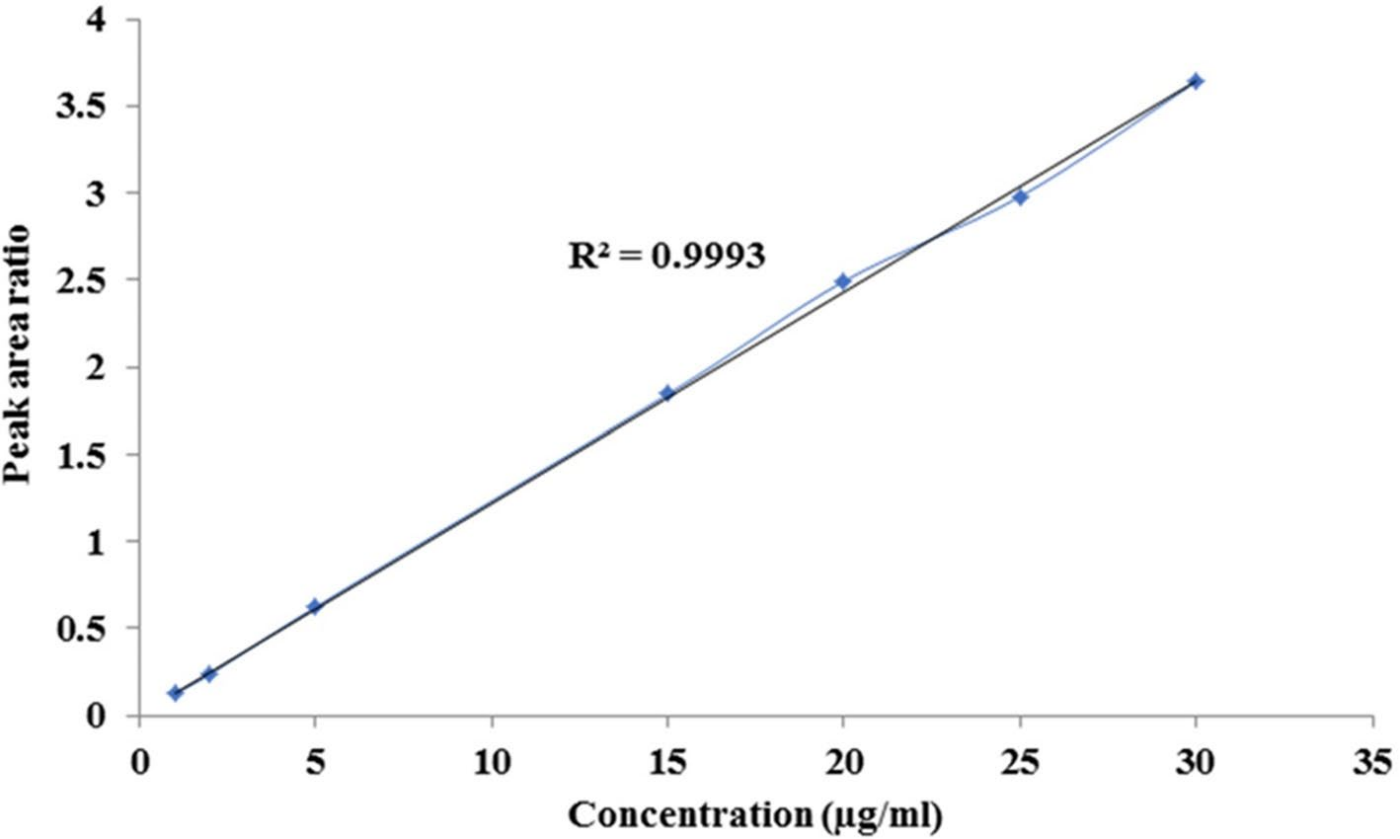
Calibration curve of atorvastatin

For the analysis of drug the calibration curve was prepared using 8 concentrations within range of 1 μg/ml to 30 μg/ml of atorvastatin calcium. The internal standard was added in each samples and peak area ratio were plotted against concentration (Fig. 4). Calibration curve was found linear in the calibration range with regression factor R^2^ = 0.9993.

### Dissolution test

Dissolution study has become an important method for testing drug release and batch to batch variation [37]. In order for a drug to be bioavailable, it must dissolve from its solid dosage form, especially for poorly soluble drugs such as atorvastatin. Poorly soluble drugs do not have adequate dissolution profiles (the first step determining the rate of absorption), so they will not be as readily available in the body system for the desired therapeutic effect. The dissolution studies may be useful for determining the amount of drug available for absorption after oral administration [24, 38].

**Fig. 5:**
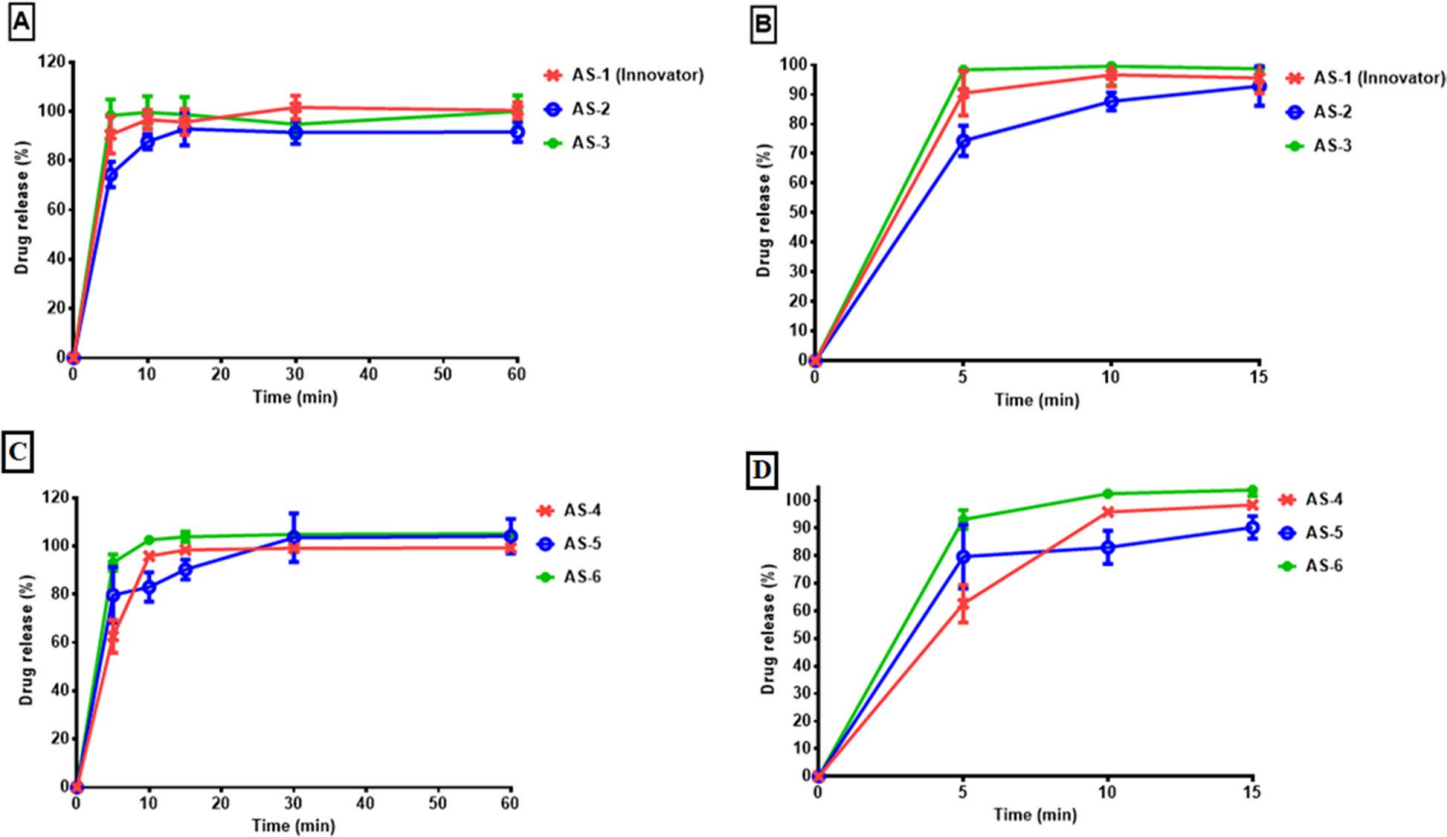
(**A**) Dissolution profiles of the atorvastatin tablets (AS-1, Innovator) and its generic counterparts (AS-2, AS-3) at pH 6.8, (**B**) Showing drug release of AS-1, AS-2, and AS-3 at 15 min. (**C**) Dissolution profiles of the atorvastatin tablets (AS-4, AS-5, and AS-6) at pH 6.8, (**D**) Showing drug release of AS-4, AS-5, and AS-6 at 15 min

The results of dissolution test exhibited that the products AS-1, AS-3, and AS-4 shows 100.44%, 100.04% and 99.25% drug release respectively within 60 min of dissolution study (Fig. 5). While product AS-2 shows 91.69% (Fig. 5). After 60 min, products AS-5 and AS-6 presented drug release of 104.09% and 105.16% respectively, product AS-6 showed the highest percentage (105.16%) of drug release and product AS-2 shows the lowest percentage of drug release (91.69%). Every one of the tested brand of tablet demonstrated fast dissolution and releasing more than 85% of the atorvastatin content in less than 15 min. In order to grant a generic a marketing permit, regulatory authorities demand evidence of similarity among the generic and innovator brands. Very rapid dissolving tablets are considered essentially similar without a need for similarity (*f*_2_) and difference (*f*_1_) factors [39, 40].

## Conclusion

The outcomes of present study revealed that, all the investigated generics brands of atorvastatin tablet (20 mg) are of good pharmaceutical quality and are comparable with the innovator product. The dissolution profile displayed that the innovator product and all the evaluated generic brands tablets disintegrated rapidly and presented more than 85% of the drug released in less than 15 minutes. The findings of present study show that all the generic brands are equivalents to innovator product with respect to the in vitro drug release features and hence these products could be used interchangeably. In general, generic version of medicines is less costly than innovator product. Hence moving to generic prescriptions will contribute to the noteworthy cost saving for the patients.

## Data Availability

All data generated or analyzed during this study are included in this published article.
